# Brothers and sisters of childhood acute leukemia survivors: Their long‐term quality of life and its determinants

**DOI:** 10.1002/cam4.5355

**Published:** 2022-10-20

**Authors:** Cindy Faust, Pascal Auquier, Zeinab Hamidou, Yves Bertrand, Marie‐Dominique Tabone, Sophie Ansoborlo, André Baruchel, Virginie Gandemer, Jean‐Hugues Dalle, Pascal Chastagner, Justyna Kanold, Maryline Poirée, Nicolas Sirvent, Geneviève Plat, Isabelle Pellier, Gérard Michel, Julie Berbis

**Affiliations:** ^1^ UR 3279, CERESS – Health Service Research and Quality of Life Center Aix‐Marseille University Marseille France; ^2^ Department of Pediatric Hematology‐Oncology University Hospital of Lyon Lyon France; ^3^ Department of Pediatric Hematology‐Oncology A. Trousseau Hospital, AP‐HP Paris France; ^4^ Department of Pediatric Hematology‐Oncology University Hospital of Bordeaux Bordeaux France; ^5^ Department of Pediatric Hematology‐Oncology Saint Louis Hospital, AP‐HP Paris France; ^6^ Department of Pediatric Hematology‐Oncology University Hospital of Rennes Rennes France; ^7^ Department of Pediatric Hematology Robert Debré Hospital, AP‐HP Paris France; ^8^ Department of Pediatric Hematology‐Oncology Children's Hospital of Brabois Vandoeuvre Les Nancy France; ^9^ Department of Pediatric Hematology‐Oncology, CIC Inserm 501 University Hospital of Clermont‐Ferrand Clermont‐Ferrand France; ^10^ Department of Pediatric Hematology‐Oncology University Hospital L'Archet Nice France; ^11^ Department of Pediatric Hematology‐Oncology University Hospital of Montpellier Montpellier France; ^12^ Department of Pediatric Hematology‐Oncology University Hospital of Toulouse Toulouse France; ^13^ Department of Pediatric Hematology‐Oncology University Hospital of Angers Angers France; ^14^ Department of Pediatric Hematology‐Oncology Timone Children's Hospital and Aix‐Marseille University Marseille France

**Keywords:** childhood leukemia, long‐term follow‐up, quality of life, siblings, survivors

## Abstract

**Background:**

Childhood cancer confront the whole family with a traumatic event. Because brothers and sisters may encounter emotional problems that can remain for a long time and that only few studies have assessed their long‐term outcome, our present objectives were to describe the long‐term quality of life (QoL) of childhood leukemia survivors' siblings and to explore its determinant.

**Methods:**

Brothers and sisters (from 8‐year‐old) of survivors included in the French LEA Cohort completed a QoL questionnaire (according to their age). Scores were compared with those reported by age‐ and gender‐matched French general population and by survivors. Using a clustering method, siblings were categorized into 3 groups depending on their level of QoL's scores and factors likely to be linked with these clusters were explored with multivariate analyses.

**Results:**

We included 689 brothers and sisters (313 minors, 376 adults) and the mean time from diagnosis was 13.2 ± 6.6 years. Minor siblings reported higher QoL scores than general population (*p* < 0.001), but a lower score for relationship with family than survivors (*p* < 0.001). In adult siblings, Mental Component Summary score was lower than general population (*p* < 0.001). Level of siblings' QoL was linked with female gender, but no association was found with cancer‐related factors.

**Conclusion:**

Brothers and sisters expressed a divergent perception of their long‐term QoL depending on their age. To minimize the impact from childhood to adulthood, long‐term attention should also be paid to siblings, often referred as “forgotten children”.

## Graphical abstract

Years after diagnosis, brothers and sisters reported a different perception of their Quality of Life (QoL) according to their age, suggesting a deterioration in adulthood compared to their peers. Being female was associated with the lowest QoL level, but no association was found with cancer‐related factors.

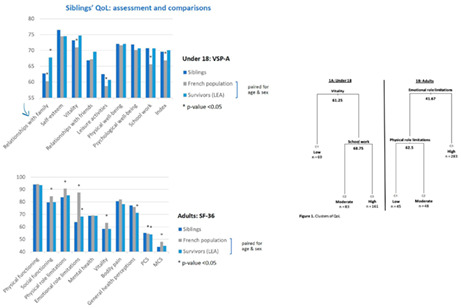

## 1 INTRODUCTION

Childhood cancer is a major cause of death worldwide and data indicates leukemia as the most common cancer type in children and teens, accounting for almost 1 out of 3 cancers.^1^ In recent decades, therapeutic innovations have increased the survival rate to approximately 80%^2^ and survivors commonly benefit from a long‐term follow‐up to watch repercussions of the disease. Indeed, there have been multiple researches on late physical side effects^3^, ^4^ and on the quality of life (QoL)^5^, ^6^ of children and adolescents with cancer, in particular from acute leukemia (AL).^7^, ^8^

Beyond the impact on survivors' life, childhood cancer is a traumatic event which also affect both parents^9^ and siblings.^10^, ^11^, ^12^ During the period of treatment, literature reported that parents' distress and their reduced availability is likely to induce higher demands on brothers and sisters to take on new daily responsibilities, possibly leading to psychosocial difficulties and specific adaptation.^13^ Indeed, some studies reported long‐term psychological repercussions^14^, ^15^ and persistent symptoms of post‐traumatic stress disorder,^16^ supporting the hypothesis of a long‐term impact on brothers and sisters. However, very few studies have been conducted on more diversified domains such as siblings' QoL and to our knowledge, the sparse literature showed substantial methodological limitations. First, most studies include siblings as a control group for survivors and not as a population of interest,^17^, ^18^, ^19^ commonly using parent proxy assessments^20^ or interviews reviewing perspectives of all the family members together.^21^ Besides, when studies' target population is brothers and sisters, samples sizes are often rather small^21^ and the time since diagnosis is quite short, frequently in the 5 years after diagnosis.^11^, ^21^ Furthermore, despite the interest in QoL assessment in clinical research having increased, so far, less effort has been made to interpret QoL data. Certainly, by translating QoL score results into meaningful and relevant categories, they would allow a more appropriate approach in clinical practice.^22^

Thus, further consideration is required to assess the consequences long after this major life event on the QoL of childhood cancer survivors' siblings. Even long after diagnosis and according to their age, the way siblings evaluate the impact of the disease on their QoL might significantly differ depending on whether the siblings are under 18 and still living at home, or if they are adults and independent by the time of the evaluation and it seems relevant to explore these two different populations.

The present objectives of this study are to compare long‐term QoL of both under 18 (child and adolescent) and adult siblings of childhood leukemia survivors with age‐ and gender‐ matched general population and then to contrast it with survivors' QoL. Additionally, we aim to create clusters of their QoL levels to determine profiles of siblings and to identify characteristics (sociodemographic, health‐related and cancer‐related factors) which can affect long‐term QoL.

## 2 MATERIALS AND METHODS

### 2.1 Subjects

The Leucémies de l'Enfant et de l'Adolescent (LEA) study is a prospective, multicenter program aiming to follow‐up at long‐term all childhood leukemia survivors treated since 1980 in France. Patients' participation starts 1 year after finishing treatment and evaluations are repeated periodically every 2 or 4 years according to their current age and to the time from diagnosis or last relapse. During specific, regular, and standardized medical visits, information like anamnesis and physical long‐term side effects are gathered while auto‐questionnaires providing further information such as QoL and socio‐occupational data are completed. According to the French law, the LEA protocol was approved by a research ethics committee and additional details about the program are available at www.plateforme‐lea.fr.^23^

In 2017, we assessed the QoL of the brothers and sisters of patients enrolled in one of the 13 participating pediatric hematology‐oncology centers of the LEA cohort. For this study, families who reported having any siblings or half siblings living together at diagnosis were considered eligible and contacted by letter. Siblings were included when following inclusion criteria were fulfilled: (a) aged 8 years minimum at the time of assessment, (b) being the sibling closest in age to the childhood AL survivor, and (c) permission to participate in the study, with consent from parents or legal guardians for any participant under 18. Siblings who did not indicate their date of birth or their gender, who answered the wrong age‐related questionnaire or did not answer the QoL questionnaire were considered as non‐participant for the study. Also, siblings were not enrolled if the time between this assessment and the survivor's last evaluation from the LEA follow‐up program exceeded 4 years.

### 2.2 Quality of life

#### 2.2.1 Under 18

To self‐assess minor siblings' QoL, two versions of the Vécu et Santé Perçue de l'Adolescent et l'enfant (VSP‐A) questionnaire were used; one validated for children aged 8 to 10 years old and one validated for adolescents up to 17 years old.^24^, ^25^, ^26^ Answers to both VSP‐A forms define eight domains of QoL and an overall score: psychological well‐being, physical well‐being, self‐esteem, vitality, leisure, relationship with friends, relationship with parents, and schoolwork. Scores scale from 0 to 100, so that higher scores indicate better QoL.

Reference values from the French general population are available for age‐ and gender‐matched comparison.^27^

#### 2.2.2 Adults

The generic self‐report 36‐item Short Form Health Survey (SF‐36) was used to assess the adult siblings' QoL. This questionnaire is validated in French^28^ and measures eight subscales of QoL, namely: physical functioning (PF), bodily pain (BP), role limitations due to physical problems (RP), role limitations due to emotional problems (RE), mental health (MH), social functioning (SF), vitality (VT) and general health perceptions (GH). Two summary measures are also provided: the physical (PCS) and the mental (MCS) component summary scores. Scores scale from 0 to 100, so that higher scores indicate better QoL.

Reference values from the French general population are available for age‐ and gender‐matched comparison.^29^

### 2.3 Factors

Several factors regarding brothers or sisters and survivors were also documented.

#### 2.3.1 Sociodemographic factors

Gender and age at evaluation were reported for both siblings and survivors. The socioeconomic situation during the treatment period, indicating financial problems in families, is documented in the LEA cohort and has been assessed via a self‐reported question, using a 5‐point Likert scale, with following answer choices “very at ease,” “at ease,” “medium,” “difficult,” and “very difficult.”

#### 2.3.2 Health‐related factors

Health status was either self‐reported for brothers and sisters or documented during medical visits implemented in the LEA cohort for survivors and referring to the late physical effects.

#### 2.3.3 Cancer‐related factors

Age at the time of diagnosis were reported for both siblings and survivors. Clinical data about the AL and its treatments were detailed in the LEA study as follows: subtype of leukemia, relapse occurrence, use of central nervous system (CNS) irradiation, total body irradiation (TBI), and hematopoietic stem cell transplantation (HSCT). Besides, we estimated an indicator interpreting the overall burden of the AL as follows: relapse (1 point), irradiation (central nervous system or total body irradiation, 1 point), and HSCT (1 point), resulting in a 3‐point rating scale.

### 2.4 Statistical analysis

Categorical variables are presented as numbers and percentages, while quantitative data is reported using mean ± standard deviation (*SD*). Within the eligible siblings, we evaluated our sample representativeness by comparing participants' and non‐participants' demographic and cancer‐related factors, using *χ* ^2^ test and Student's *t*‐test (for percentages and means, respectively).

For both minor and adult siblings, QoL mean scores for each dimension were computed using the scoring algorithms made accessible by the questionnaires' inventors. We compared siblings' scores with (a) the French general population using paired Student t‐tests, according to siblings' age and gender, (b) AL survivors' scores using a GLM multivariate analysis of variance (MANOVA), with siblings' age, gender and time since diagnosis as covariates. Effect sizes, representing here the magnitude of the difference in changes between two groups, were obtained by dividing the difference in means by the reference *SD*, to present a clearer interpretation of health status outcomes and whether these changes are clinically relevant. A difference was considered meaningful whereby 0.2 equates to a small effect, 0.5 represents a moderate effect size and 0.8 a large effect size.^30^, ^31^

Siblings were categorized using a top‐down hierarchical clustering method according to their level of QoL. A three‐group structure was considered and the minimum number of siblings in each cluster was constrained to be 25. During the procedure of the hierarchical top‐down method of Clustering using Unsupervised Binary Trees (CUBT), a maximal tree is first developed; then is pruned using a minimal dissimilarity criteria while paying attention if adjacent nodes can be aggregated; and finally, the most identical clusters were combined together.^22^, ^32^ Scores of the 8‐dimension of the VSP‐A were simultaneously used to categorize each minor sibling into one cluster based on their QoL level, whereby the upper cluster represented siblings with the highest QoL scores, the medium cluster to moderate QoL scores and the low one to the lowest QoL scores. The same procedure was performed for adults' siblings using the SF‐36 questionnaires.

For both age categories of siblings, we compared these clusters with sociodemographic, health‐ and cancer‐related factors. Multinomial logistic regression models, adjusted for siblings' age and gender, were estimated to determine characteristics that might predict brothers' and sisters' QoL levels. Selection of relevant variables for the models was made within univariate analyses when results of analysis of variance or chi‐squared tests provided *p* ≤ 0.25. Post hoc analysis was performed to identify differences between each category of QoL level.

All tests were two‐sided and *p*‐value threshold for statistical significance was set to 5% (or 0.05). IBM SPSS Statistics version 20.0 (SPSS Inc.) and the CUBT R package version 1.0.26 were both used to compute statistical analyses.

## 3 RESULTS

### 3.1 Participants

**FIGURE 1:**
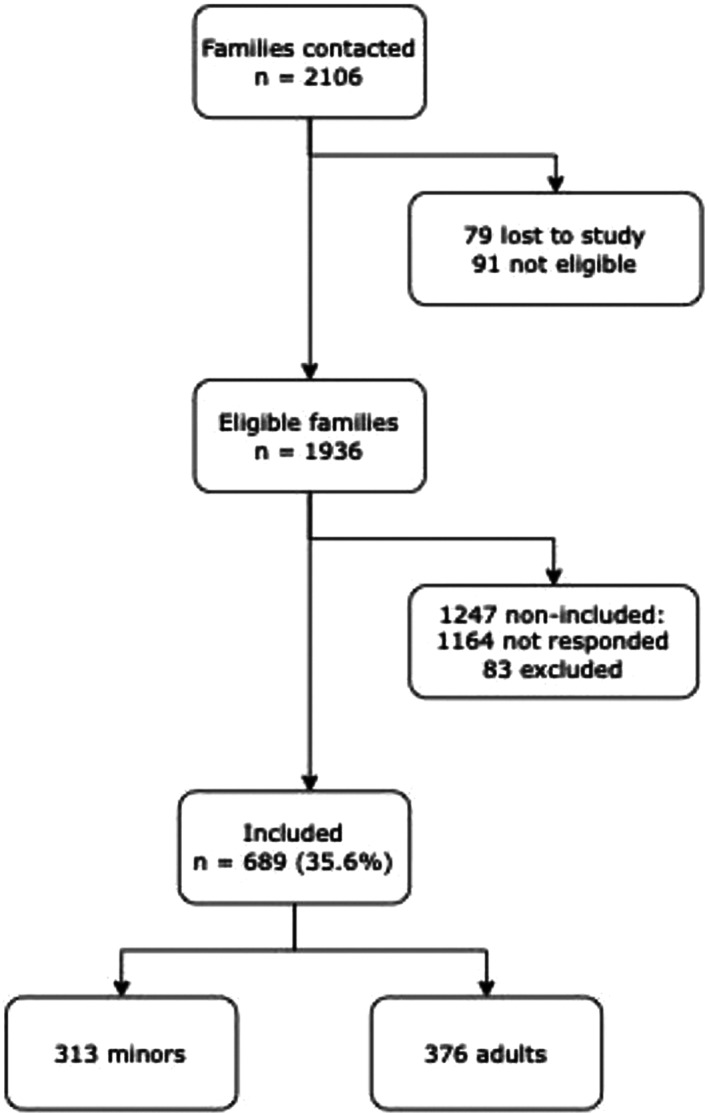
Flowchart

Of the 2106 participant families listed in the centers of LEA cohort in 2017, 79 were considered lost to the study and for an extra 91, brothers or sisters did not fulfill the inclusion criteria. By all the 1936 eligible brothers and sisters, 689 completed the questionnaire (response rate 35.6%) including 376 adults and 313 minors, as in the flowchart shown in Figure 1.

Comparison of eligible families based on cancer‐related factors faced by the family is provided in Table 1.

**TABLE 1:** Comparison of participant and non‐participant groups

	Under 18 *n* = 313	Adults *n* = 376	Study population *n* = 689	Non‐included population *n* = 1247	*p* values
**Survivor's characteristics**					
Age at diagnosis (*μ* ± *σ*)	4,6 ± 2,7	7,3 ± 4,7	6.0 ± 4.1	6.8 ± 4.5	**<0.001**
Sex					0.14
Male	162 (51.8)	174 (46.3)	336 (48.8)	653 (52.4)	
Female	151 (48.2)	202 (53.7)	353 (51.2)	594 (47.6)	
Type of acute leukemia					0.95
Lymphoblastic	275 (87.9)	318 (84.6)	592 (85.9)	1073 (86.0)	
Myeloblastic	38 (12.1)	58 (15.4)	97 (14.1)	174 (14.0)	
Relapse					0.33
Yes	27 (8.6)	44 (11.7)	71 (10.3)	148 (11.9)	
No	286 (91.4)	332 (88.3)	618 (89.7)	1099 (88.1)	
HSCT					0.63
No	274 (87.5)	287 (76.3)	561 (81.4)	1000 (80.2)	
Yes without TBI	18 (5.8)	35 (9.3)	53 (7.7)	93 (7.5)	
Yes with TBI	21 (6.7)	54 (14.4)	75 (10.9)	154 (12.3)	
CNS Irradiation					**0.007**
Yes	13 (4.2)	31 (8.3)	44 (6.4)	130 (10.5)	
No	300 (95.8)	344 (91.7)	645 (93.6)	1117 (89.5)	
No. of late physical effect					0.70
0 or 1	202 (64.5)	187 (49.7)	389 (56.5)	692 (55.5)	
≥2	111 (35.5)	189 (50.3)	300 (43.5)	555 (44.5)	
Burden index of leukemia					0.12
0–1	282 (90.1)	313 (83.2)	595 (86.4)	1043 (83.6)	
2–3	31 (9.9)	63 (16.8)	94 (13.6)	204 (16.4)	

*Note*: Bold values: *p*‐value <0.05 was significant.

Abbreviations: CNS, central nervous system; HSCT, hematopoietic stem cell transplantation; TBI, total body irradiation; *μ*, mean; *σ*, standard deviation.

Participant and non‐participant groups were not significantly different regarding the survivor's gender, type of leukemia, relapse status, number of late physical effects and history of transplantation with or without TBI. However, the rate of CNS irradiation was significantly higher in the non‐responder group (10.5% vs 6.4%; *p* = 0.007) and they were less than one‐year older than the study population (6.8 ± 4.5 years versus 6.0 ± 4.1 years; *p*‐value <0.001).

Descriptive characteristics of responding siblings is provided in Table 2.

**TABLE 2:** Population characteristics

	Under 18 *n* = 313	Adults *n* = 376	Total *n* = 689
**Sibling's characteristics**			
Sex			
Male	144 (54.0)	149 (39.6)	293 (42.5)
Female	169 (46.0)	227 (60.4)	396 (57.5)
Age at evaluation (*μ* ± *σ*)	13.4 ± 2.8	26.6 ± 6.6	20.6 ± 8.4
Minors			
8–10 years	80 (25.6)	—	80 (11.6)
11–17 years	233 (74.4)	—	233 (33.8)
Adults			
18–24 years	—	193 (51.3)	193 (28.0)
≥25 years	—	183 (48.7)	183 (26.6)
Age at survivor diagnosis (*μ* ± *σ*)	4.8 ± 3.0	10.7 ± 5.4	8.2 ± 5.4
Not yet born ‐ 5 years	188 (60.1)	59 (15.7)	247 (35.8)
5–10 years	113 (36.1)	128 (34.0)	241 (35.0)
≥10 years	12 (3.8)	189 (50.3)	201 (29.2)
Years since survivor diagnosis			
(*μ* ± *σ*)	9.6 ± 3.4	16.3 ± 7.1	13.2 ± 6.6
(med [min;max])	9.1 [2.3; 25.1]	15.3 [2.3; 36.7]	11.7 [2.3; 36.7]
Minors			
<10 years	185 (59.1)	—	—
≥10 years	128 (40.9)	—	—
Adults/Both			
<15 years	—	181 (48.1)	476 (69.1)
≥15 years	—	195 (51.9)	213 (30.9)
Types of sibling^a^			
Twins	6 (2.3)	9 (2.4)	15 (2.4)
Brothers & Sisters	247 (94.6)	352 (93.6)	599 (94.0)
Half siblings	8 (3.1)	15 (4.0)	23 (3.6)
Sibling older			
No	178 (56.9)	92 (24.5)	270 (39.2)
Yes (or twins)	135 (43.1)	284 (75.5)	419 (60.8)
Donor			
Yes	9 (2.9)	37 (9.8)	46 (6.7)
No	304 (97.1)	339 (90.2)	643 (93.3)
Chronic diseases^b^			
Yes	30 (11.6)	53 (14.2)	83 (13.2)
No	229 (88.4)	319 (85.8)	548 (86.8)
**Family's characteristics**			
Financial situation^c^			
At ease or medium	216 (90.8)	242 (88.0)	458 (89.3)
Difficult	22 (9.2)	33 (12.0)	55 (10.7)

Abbreviations: *μ*, mean, *σ*, standard deviation.

^a^ Missing data: *n* = 52 (minors).

^b^ Missing data: *n* = 75 (minors) *n* = 101 (adults).

^c^ Missing data: *n* = 75 (minors) *n* = 101 (adults).

Overall, the mean time since diagnosis was 13.2 ± 6.6 years. In siblings aged under 18, this mean time was 9.6 ± 3.4 years with a mean age at the time of assessment of 13.4 ± 2.8 years. Among adult siblings, the mean time since diagnosis was 16.3 ± 7.1 years and the mean age at evaluation was 26.6 ± 6.6 years. Finally, most siblings (86.8%) did not report any chronic diseases.

### 3.2 Long‐term QoL in brothers and sisters of childhood AL survivors

#### 3.2.1 Siblings under 18

Siblings showed the lowest score in the leisure domain and the highest score in the self‐esteem domain, similarly to those of the French general population (Table 3A).

**TABLE 3:** Quality of life measurements in minors siblings (A) and in in adults siblings (B) compared with French reference population and leukemia survivors

VSP‐A	Siblings (*n* = 313)	French norms					Survivors				Effect size (ranking)
		Expected data^a^	Difference		*p* values	Effect size (ranking)	LEA data^b^ (*n* = 345)	Difference		*p* values	
			Mean ± *SD*	95% CI				Mean ± SD	95% CI		
**(A)**											
Relationships with family	62.72 ± 22.65	60.26	2.47 ± 21.43	**0.08**; **4.85**	**0.04**	0.12 (4)	67.76 ± 20.95	−5.41 ± 21.90	−**8.69**; −**2.13**	**0.001**	−0.25
Self‐esteem	76.50 ± 24.98	74.48	2.02 ± 23.94	−0.64; 4.69	0.14		74.52 ± 27.05	2,37 ± 26.08	−1.48; 6.22	0.23	
Vitality	73.19 ± 17.97	70.96	2.24 ± 17.26	**0.32**; **4.16**	**0.02**	0.13 (3)	74.72 ± 17.54	−1,49 ± 17.75	−4.10; 1.12	0.26	
Relationships with friends	66.88 ± 21.79	67.20	−0.33 ± 20.95	−2.66; 2.00	0.78		69.56 ± 20.45	−2,03 ± 21.13	−5.17; 1.11	0.21	
Leisure activities	62.50 ± 18.31	58.80	3.71 ± 18.44	**1.66**; **5.76**	**<0.001**	0.20 (2)	60.72 ± 21.01	1,81 ± 19.78	−1.22; 4.84	0.24	
Physical well‐being	72.06 ± 18.31	71.66	0.40 ± 18.02	−1.60; 2.41	0.69		72.05 ± 19.24	0.32 ± 18.79	−2.51; 3.15	0.82	
Psychological well‐being	71.89 ± 20.44	70.14	1.76 ± 19.53	−0.42; 3.93	0.11		70.67 ± 21.37	1,68 ± 20.93	−1.44; 4.81	0.29	
School work	70.73 ± 22.54	65.58	5.15 ± 22.95	**2.59**; **7.70**	**<0.001**	0.22 (1)	70.65 ± 22.52	−0.63 ± 22.51	−4.01; 2.76	0.72	
Index	69.56 ± 12.78	66.82	2.74 ± 12.54	**1.35**; **4.14**	**<0.001**		70.08 ± 12.49	−0.42 ± 12.62	−2.30; 1.45	0.66	

SF‐36	Siblings (*n* = 376)	French norms					Survivors				
		Expected data^a^	Difference		*p* values	Effect size (ranking)	LEA data^b^ (*n* = 281)	Difference		*p* values	Effect size (ranking)
			Mean ± *SD*	95% CI				Mean ± *SD*	95% CI		
**(B)**											
Physical functioning	94.18 ± 13.83	94.48	−0.30 ± 13.81	−1.70; 1.10	0.67		93.70 ± 12.92	1,14 ± 13.44	−0.97; 3.26	0.29	
Social functioning	79.55 ± 22.81	84.55	−4.99 ± 22.78	−**7.30**; −**2.68**	**<0.001**	−0.22 (4)	79.85 ± 22.42	−0.25 ± 22.63	−3.79; 3.29	0.89	
Physical role limitations	83.69 ± 29.03	90.62	−6.93 ± 29.03	−**9.87**; −**3.99**	**<0.001**	−0.24 (3)	85.29 ± 27.83	−1,34 ± 28.51	−5.83; 3.16	0.56	
Emotional role limitations	63.79 ± 29.39	87.48	−23.70 ± 29.15	−**26.65**; −**20.74**	**<0.001**	−0.81 (1)	68.27 ± 27.57	−5,21 ± 28.69	−**9.75**; **0.68**	**0.02**	−0.18
Mental health	68.94 ± 17.45	69.19	−0.24 ± 17.56	−2.02; 1.54	0.80		68.98 ± 17.19	0.05 ± 17.33	−2.73; 2.63	0.97	
Vitality	58.47 ± 18.72	63.23	−4.77 ± 18.81	−**6.67**; −**2.86**	**<0.001**	−0.25 (2)	58.41 ± 18.17	0.23 ± 18.47	−2.61; 3.08	0.87	
Bodily pain	80.63 ± 22.20	82.01	−1.38 ± 22.21	−3.63; 0.88	0.23		78.26 ± 22.41	2,71 ± 22.31	−0.77; 6.19	0.13	
General health perceptions	77.16 ± 17.33	75.96	1.20 ± 17.18	−0.55; 2.94	0.18		71.20 ± 21.32	7,01 ± 19.35	**4.05**; **9.97**	**<0.01**	0.36
Physical composite score (PCS)	55.19 ± 6.44	54.50	0.70 ± 6.34	**0.05**; **1.34**	**0.03**		53.94 ± 7.55	1,63 ± 6.96	**0.54**; **2.71**	**0.003**	
Mental composite score (MCS)	44.02 ± 10.27	48.01	−3.99 ± 10.33	−**5.03**; −**2.94**	**<0.001**		44.78 ± 9.63	−0.93 ± 10.00	−2.49; 0.62	0.24	

*Note*: Bold values: *p*‐value <0.05 was significant.

Abbreviations: CI, confidence interval; *SD*, standard deviation.

^a^ Age and sex macthed.

^b^ Age, sex and time since diagnosis matched.

Compared to this general population, siblings expressed better QoL scores in most of domains of the VSP‐A questionnaire with significant differences but insignificant effect sizes going from 0.12 to 0.22 in the following areas: schoolwork, leisure activities, vitality and relationship with family. Overall, siblings had significantly higher VSP‐A index score than the general population (*p*‐value <0.001).

On the contrary, when compared with childhood AL survivors' QoL after adjustment for age, gender and years since diagnosis, no significant differences were found except “relationship with family” score, which was significantly lower for siblings: 62.7 ± 22.7 versus 67.8 ± 21.0.

#### 3.2.2 Adult siblings

Brothers and sisters of childhood leukemia survivors, similarly to the age‐ and gender‐ matched French general population, reported the lowest score in the vitality domain and the highest score in the physical functioning domain (Table 3B).

In comparison with the general population, siblings' QoL scores were significantly lower for 4 of the 8 dimensions of the SF‐36, with the largest effect size for emotional role functioning (0.81), followed by vitality, physical role, and social life, respectively ranging from 0.25 to 0.22. The MCS score was also significantly lower for siblings than for the general population.

Compared with leukemia survivors' QoL, siblings reported significantly lower emotional role functioning and a significant higher perception of their general health and PCS score.

### 3.3 Clusters of siblings' QoL levels and their determinants

#### 3.3.1 Siblings under 18

**FIGURE 2:**
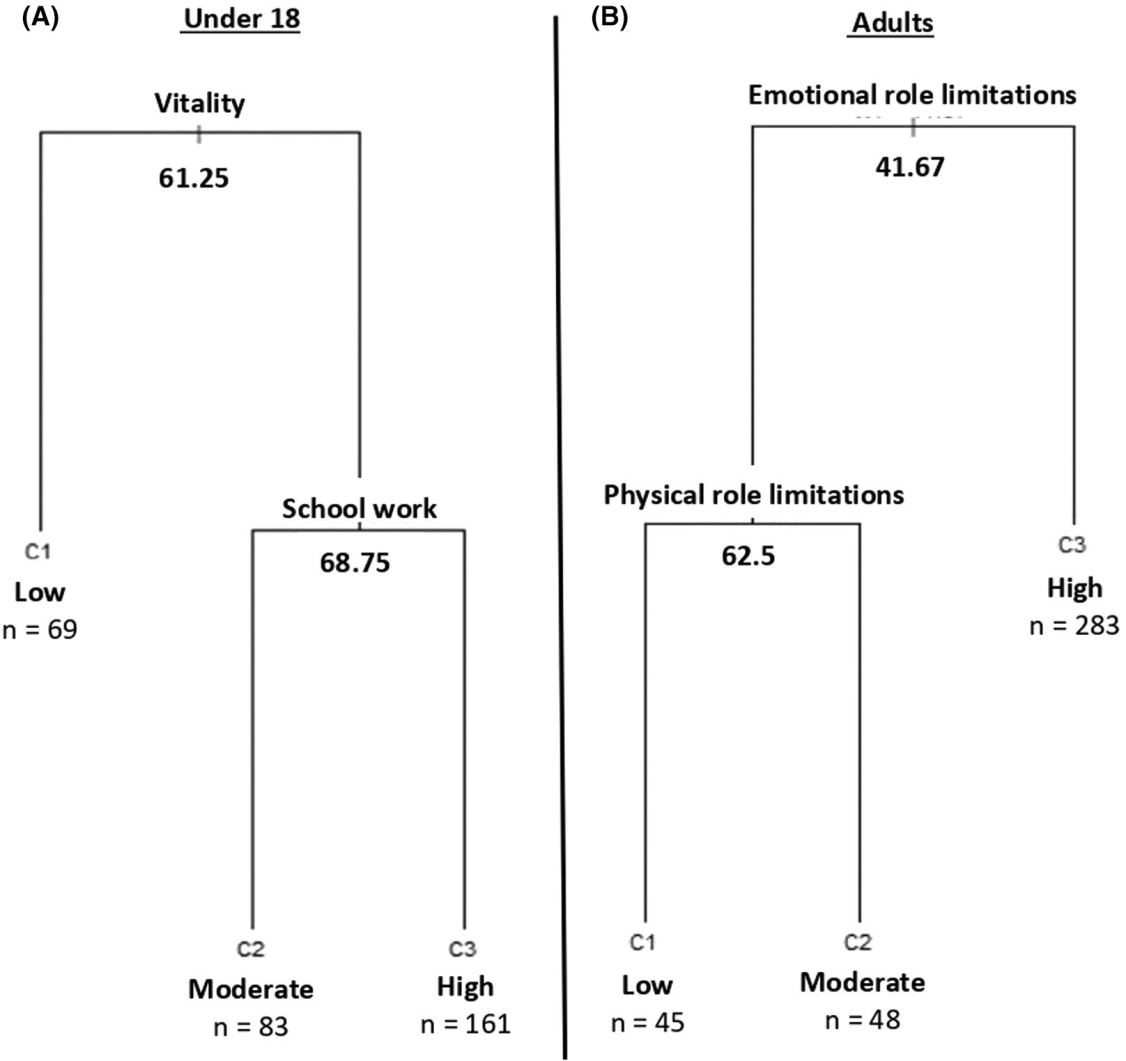
Clusters of QoL

Among the 313 minor siblings, the 3‐cluster structure classified 69 brothers and sisters with an inferior level of QoL, 83 with a moderate level, and 161 had a high level (Figure 2A). The two subscales that differentiated the siblings were vitality and schoolwork. Participants with a vitality score under 61.25 were in the low level of QoL cluster and among other siblings, those with schoolwork dimension scores over 68.75 were in the upper level of QoL cluster. Scores of all VSP‐A dimensions for each cluster are provided in Table 4A. VSP‐A index score was 53.6 ± 10.3 for siblings classified in the inferior level of QoL cluster, 68.7 ± 7.7 for siblings in the moderate cluster and 76.8 ± 8.9 for participants in the high QoL cluster.

**TABLE 4:** (A) VSP‐A dimension scores by cluster of QoL. (B) SF‐36 dimension scores by cluster of QoL

VSP‐A	Cluster 1, low QoL (*n* = 69)	Cluster 2, moderate QoL (*n* = 83)	Cluster 3, high QoL (*n* = 161)
**(A)**			
Relationships with family	45.36 ± 22.81	63.39 ± 18.09	69.82 ± 20.76
Self‐esteem	53.25 ± 30.29	80.16 ± 19.32	84.59 ± 18.15
Vitality	47.23 ± 12.71	75.93 ± 9.62	82.90 ± 11.14
Relationships with friends	52.97 ± 22.93	69.80 ± 21.39	71.33 ± 18.98
Leisure activities	53.71 ± 19.21	63.43 ± 17.84	65.79 ± 17.01
Physical well‐being	59.78 ± 17.63	73.75 ± 17.52	76.45 ± 16.68
Psychological well‐being	52.71 ± 22.05	75.81 ± 13.71	78.09 ± 17.50
School work	64.13 ± 22.68	47.14 ± 14.65	85.71 ± 11.34
Index	53.64 ± 10.33	68.68 ± 7.72	76.84 ± 8.91

SF‐36	Cluster 1, low QoL (*n* = 45)	Cluster 2, moderate QoL (*n* = 48)	Cluster 3 high QoL (*n* = 283)
**(B)**			
Physical functioning	87.65 ± 15.50	94.06 ± 13.93	95.23 ± 13.29
Social functioning	48.06 ± 19.94	64.06 ± 19.74	87.19 ± 17.53
Physical role limitations	23.89 ± 21.29	94.27 ± 10.62	91.40 ± 19.54
Emotional role limitations	17.78 ± 16.82	23.61 ± 15.31	77.92 ± 15.88
Mental health	48.89 ± 16.56	56.56 ± 13.87	74.23 ± 14.55
Vitality	38.69 ± 16.43	48.73 ± 17.78	63.26 ± 16.43
Bodily pain	59.96 ± 24.19	81.40 ± 20.89	83.79 ± 20.33
General health perceptions	61.93 ± 20.24	73.44 ± 16.62	80.21 ± 15.51
Physical Composite Score (PCS)	49.02 ± 6.96	60.15 ± 5.96	55.33 ± 5.60
Mental Composite Score (MCS)	30.36 ± 7.08	32.06 ± 8.32	48.22 ± 7.00

Of all the explored characteristics, we found that only sociodemographic factors were still associated with the low level of QoL cluster in the multivariate analysis (Supplementary File 1). Girl siblings were significantly predominant in the lowest QoL level (*p* = 0.01) whereas the youngest brothers and sisters (less than 10 years old) were significantly prevalent in the high QoL cluster (*p* = 0.02 compared to the inferior cluster).

#### 3.3.2 Adult siblings

Clustering results assigned 376 siblings of childhood AL survivors as follows: 45 had a low level of QoL, 48 a moderate, and 283 a high level (Figure 2B). Illustration of the 3‐cluster structure indicated that siblings were initially distinguished by their score in the “emotional role limitations” domain, which classified siblings in the upper QoL cluster when it exceeded 41.67. Other siblings were classified in the low QoL cluster when their “physical role limitations” score was under 62.5. Table 4B reported SF‐36 scores for each component according to the clusters. Regarding the two summary scales (PCS and MCS), participants classified in the upper QoL cluster got scores of 55.3 ± 5.6 and 48.2 ± 7.0, those in the moderate cluster scored 60.2 ± 6.0 and 32.1 ± 8.3, and siblings in the low cluster got 49.0 ± 7.0 and 30.4 ± 7.1, respectively.

Results of the univariate and multivariate analyses showed that siblings' gender and AL survivor's gender were linked with siblings' QoL levels (Supplementary File 2). Indeed, being a woman (*p* = 0.03) and having a sister as survivor (*p* = 0.01) were two determinants of an inferior level of QoL. However, reporting a chronic disease was no longer a significant factor in the multivariate analysis (*p* = 0.02 vs. *p* = 0.07).

## 4 DISCUSSION

Years after diagnosis, minor siblings reported overall scores in QoL that were very similar to the general population but also to those of AL survivors. However, adult siblings, especially when compared with the general population, reported worse QoL scores in all components of the QoL: physical, social, and mental domains. While scientific literature often concurred with siblings' psychosocial consequences, our findings suggest that, long after the remission of cancer, adult sibling QoL is impaired in every domain and especially the “emotional role limitation” scale, which measures limitation occurring at work or during other activities because of mental health difficulties.

Thus, depending on the siblings' age category, our results highlighted several differences. Indeed, when they were still under 18, cancer seemed to have less negative long‐term effects on perceived minor siblings' overall QoL and results are consistent with those of AL survivors^33^ and also with a previous pilot study from our cohort^34^ where siblings' VSP‐A index score was higher than those of the general population. A possible explanation for these outcomes could be their young age and their capacity to cope and to deal with difficult events during their childhood. Younger siblings might have been given coping strategies improving their resilience and minimizing the repercussion of cancer on their QoL.

On the contrary, self‐reported QoL among adult siblings seemed to be negatively affected. A study from the CCSS reported similar results about vitality (life satisfaction, happiness, self‐confidence and being in good spirits) being significantly altered compared with the general population.^35^ The negative dynamics we observed in older siblings may suggest that coping strategies implemented during childhood have reached their limit and are no longer sufficient when siblings have to face challenges of adult life and they become less carefree. Issues of the transition between childhood and adulthood have been documented in patients suffering from chronic diseases, particularly in oncology, but no studies have explored this phenomenon within the siblings' population. This statement reinforces our aim to give them particular attention and to identify the profile of siblings for whom the transition is more complicated and consequently, negatively affecting their long‐term QoL.

Nonetheless, among minor siblings, some of our findings should encourage additional research to explore the long‐term impact of cancer on specific dimensions of their QoL.

Firstly, in comparison with AL survivors, siblings' score in the “relationship with family” domain was significantly lower. During the period of treatment, previous studies revealed that siblings experienced disruptions in their family life and therefore they could have worse family relationships than patients.^12^ Indeed, a systematic review relating siblings' of children with cancer lives^10^ reported that they often faced a loss of their daily routines, a feel of being neglected in their families and a degraded relationship with their sick brother or sister. Similarly, a recent qualitative study^11^ related that most siblings sensed that their family had been torn apart, leading to an increased focus on the sick child and a change in family roles and responsibilities. Therefore, it seems that the family functioning perceived by minor siblings may still be impaired at long term and so it is important to understand its mechanism to support each family during cancer, but also after recovery.

Secondly, results from the clustering procedure implied that the siblings' perception of “schoolwork” had a negative impact on their global QoL. Scientific literature regarding sibling's education has been mainly explored at short term and findings are rather contradictory. A recent study^36^ related that brothers and sisters kept going to school as usual and that their grades were unaffected. However, these findings especially applied to those who were already academically strong. Meanwhile, other studies reported that parents' attempts to handle schedules jeopardized the siblings' school attendance, that the diagnosis created difficulties in focusing at school for siblings and, as a result they encountered academic and social difficulties.^13^, ^37^ These outcomes reported during the acute phase could suggest that, at long term and even when their brother or sister survived cancer, repercussions of this traumatic event may persist and continue to negatively influence siblings' perception of their schoolwork.

Our results about characteristics linked with siblings' QoL showed no association between cancer‐related factors and the 3 clusters of QoL levels, regardless of siblings' age categories. These outcomes are consistent with previous studies since the burden of cancer, relapse or treatment were not associated with impaired QoL, according to a report from the CCSS about sibling distress.^38^ Beyond the medical aspect, the whole cancer experience is a traumatic event, and individual or family characteristics are probably the main factors affecting the QoL. Therefore, among the characteristics we explored in siblings, we found that being older and being a girl/woman or having a sister as an AL survivor were linked with a low QoL perception. While the first affirmation about siblings' age emphases our hypothesis about the difficulties of the transition from childhood to adulthood, our results about their gender attribute are consistent with a review of the literature in brothers and sisters of children with chronic disease,^39^ but also from a recent study about AL survivors' parents who reported that being the parent of a girl survivor or being a mother were two factors linked with an impaired long‐term parents' QoL.^40^ Overall, this may suggest a persisting concern about cancer despite the length of time since recovery, which seems to be more pronounced among females, and who probably need to be given more consideration in the long‐term support of families.

### 4.1 Limits and strengths

We recognize several limitations in our study. The specific study design provides a cross‐sectional view of the siblings' QoL, so we were not able to investigate their QoL over time. A longitudinal study would be relevant so that we can explore more accurately the dynamic and the changes our study suggested between childhood and adulthood. QoL, as a highly subjective and changing concept over time, is also widely multidimensional and therefore, can partially be explained by further factors that may fluctuate throughout life. However, resources such as the ease of access to a psychologist or the living and family environment, at the time of diagnosis but also at long term, were not available in our data collection. Another limit concerns the representativeness of our sample. First, our population only consisted of siblings of AL survivors, but their experience might be more favorable than when the patient did not survive and the impact on QoL of bereaved siblings should be explored in a dedicated study. Secondly, results from the comparison between respondents and non‐respondents showed, among survivor's cancer‐related factors, a significantly higher rate of CNS irradiation in non‐responder siblings. Nevertheless, no significant difference was found with the number of late physical effects. Finally, despite only being moderate, our response rate is consistent with literature about siblings' recruitment in clinical research.^41^ However, our sample size (n = 689 siblings) which includes 313 minors and 376 adults places our study as one of the largest studies published so far, on sibling populations. Also, being a multicenter cohort including 13 pediatric hematology‐ oncology recruitment centers, the LEA study allows geographical coverage of almost three quarters of the child survivors after leukemia diagnosed during childhood in France after 1980 and therefore it is highly representative at a national level.

The principal strength of our study is the average time since diagnosis as the overall mean was above 13 years, a much longer time than many previous studies have documented.

Furthermore, the conditions to assess siblings' QoL at long term in our study provide many advantages: (a) the use of a self‐report questionnaire assessing QoL for minor siblings, avoids the discrepancy of parent proxy‐reporting, (b) as a result of the quantity and the quality of data collected from the LEA study, we managed to explore multiple domains of QoL but especially multiple determinants including sociodemographic, health‐related and cancer‐related factors, (c) we used a new clustering method, creating clusters of high, moderate and low levels of minor and adult siblings' QoL, to enhance clinical interpretation of these measurements.

## 5 CONCLUSION

Our study assessed a population of childhood AL survivors' siblings and related that, at long term, minors and adults expressed a divergent self‐perception of QoL. Despite some difficulties identified in their relationship with family, minor siblings described similar or higher QoL scores than their peers. On the other hand, adult siblings showed a poorer QoL, in all dimensions and not only in the psychosocial domain.

In addition, results from our study contribute to enlighten characteristics of siblings who may need support and more specifically in which domains of QoL they could benefit from it, so the support system may adjust to the transition and reduce difficulties from childhood to adulthood.

Altogether and besides to the survivors, these outcomes should encourage the health service to remain attentive at long term to the family and especially to these brothers and sisters, often referred as the “forgotten children”, irrespective of their age, the family composition, or the clinical data regarding AL history and treatments.

## AUTHOR CONTRIBUTIONS

**Cindy Faust:** Formal analysis (lead); visualization (lead); writing – original draft (lead); writing – review and editing (lead). **Pascal Auquier:** Conceptualization (lead); funding acquisition (equal); investigation (equal); supervision (equal); writing – review and editing (equal). **Zeinab Hamidou:** Methodology (equal); writing – review and editing (equal). **Yves Bertrand:** Funding acquisition (equal); investigation (equal); resources (equal); writing – review and editing (equal). **Marie‐Dominique Tabone:** Funding acquisition (equal); investigation (equal); resources (equal); writing – review and editing (equal). **Sophie Ansoborlo:** Funding acquisition (equal); investigation (equal); resources (equal); writing – review and editing (equal). **Andre Baruchel:** Funding acquisition (equal); investigation (equal); resources (equal); writing – review and editing (equal). **Virginie Gandemer:** Funding acquisition (equal); investigation (equal); resources (equal); writing – review and editing (equal). **Jean‐Hugues Dalle:** Funding acquisition (equal); investigation (equal); resources (equal); writing – review and editing (equal). **Pascal Chastagner:** Funding acquisition (equal); investigation (equal); resources (equal); writing – review and editing (equal). **Justyna Kanold:** Funding acquisition (equal); investigation (equal); resources (equal); writing – review and editing (equal). **Maryline Poirée:** Funding acquisition (equal); investigation (equal); resources (equal); writing – review and editing (equal). **Nicolas Sirvent:** Funding acquisition (equal); investigation (equal); resources (equal); writing – review and editing (equal). **Geneviève Plat:** Funding acquisition (equal); investigation (equal); resources (equal); writing – review and editing (equal). **Isabelle Pellier:** Funding acquisition (equal); investigation (equal); resources (equal); writing – review and editing (equal). **Gérard Michel:** Conceptualization (lead); funding acquisition (equal); investigation (equal); supervision (equal); writing – review and editing (equal). **Julie Berbis:** Funding acquisition (equal); methodology (equal); validation (lead); writing – review and editing (equal).

## CONFLICT OF INTEREST

The authors declare no conflict of interest.

## ETHICAL STATEMENT

This study was approved by the SUD MÉDITERRANÉE Ethics Committee (reference RO – 2016/20) and informed consent was not required for this observational study.

## Supporting information


Table S5

Table S6

Data S1

## Data Availability

The data that support the findings of this study are available on request from the corresponding author.
