# Supplementary material for: Brothers and sisters of childhood acute leukemia survivors: Their long‐term quality of life and its determinants
Source: Cancer Med. 2022 Oct 20;12(5):6200–12. doi: 10.1002/cam4.5355 (PMC10028038; doi:10.1002/cam4.5355)
Supplement: Supplementary file 3 — Data S1 [file CAM4-12-6200-s003.pdf]

Members of the L.E.A. study group

Marseille: AUQUIER Pascal, BARLOGIS Vincent, BERBIS Julie, BERGEROT Astrid, BOURGUE Françoise, CHAMBOST Hervé, CURTILLET Catherine, HAMIDOU Zeinab, MICHEL Gérard, SAULTIER Paul, SHAWKET Alaa, STERIN Arthur, SZEPETOWSKI Sarah, VISENTIN Sandrine, THURET Isabelle,
Nancy: CHASTAGNER Pascal, DETRAIT M, FOUYSSAC Fanny, LEMELLE Irène, MANSUY Ludovic, NUI Laurence, PERROT Aurore, PHULPIN Aurélie, POCHON Cécile, ROTH-GUEPIN Gabrielle, RUBIO M-Thérèse, SCHMITT Claudine,
Nice: BENADIBA Joy, DUHIL de BENAZE Gwénaelle, HADZIC Biljana, MONPOUX Fabrice, POIREE Maryline, PONDROM Morgane, ROHRLICH Pierre-Simon, SOLER Christine
Clermont-Ferrand: DORE Eric, GIRARD-MONIN Pauline, GREZE Victoria, ISFAN Florentina, KANOLD Justyna, MERLIN Etienne, ROUEL Nadège,
Grenoble: ADJAOUD Dalila, ARMARI-ALLA Corinne, BOBILLOT-CHAUMONT Séverine, MARTIN M-Pierre, PAGNIER Anne, PLANTAZ Dominique,
Lyon: BERTRAND Yves, GARNIER Nathalie, HALFON-DOMENECH Carine, HU Julie-Yi, KEBAÏLI Kamila, OUACHE-CHARDIN Marie, RENARD Cécile
Paris Trousseau: AUVRIGNON Anne, BOUAYAD AGHA Latéfa, DOLLFUS Catherine, DONADIEU Jean, GOURAUD Françoise, LANDMAN-PARKER Judith, LEVERGER Guy, PELLEGRINO Béatrice, PETIT Arnaud, TABONE Marie Dominique
St Etienne: BERGER Claire, DAVID Audrey, SOLER Catherine, STEPHAN Jean Louis, THOUVENIN-DOULET Sandrine
Rennes: BAYART Sophie, BONNEAU Jacinthe, CHAPPE Céline, GANDEMER Virginie, LAMOUR Anne- Marie, PUISEUX Chloé, TAQUE Sophie, TOUTAIN Fabienne
Montpellier: AKBARALY Tasnim, ELKHATIB Nour, HAOUY Stéphanie, SAUMET Laure, SIRVENT Nicolas, SIRVENT Anne, TEYSSIER Anne-Charlotte
Paris St Louis – Robert Debré: BARUCHEL André, BRETHON Benoît, LEBLANC Thierry, MOUKOKO Marie- Noelle, RAY-LUNVEN Anne-France, AZARNOUSH Saba, DALLE Jean- Hugues, FAHD Raymonda, GRAIN Audrey, LE MOUEL Lou, YAKOUBEN Karima
Bordeaux: ALADJIDI Nathalie, ANSOBORLO Sophie, DE BOUYN-ICHER Céline, DUCASSOU Stéphane, JUBERT Charlotte, MERCHED Maria, NOTZ-CARRERE Anne, VERITE-GOULARD Cécile, WALLET Emilie,
Strasbourg: BERGTHOLD Guillaume, LUTZ Patrick, PAILLARD Catherine, VITET Ludivine,
Angers: BERARDI Elsa, BRASME Jean-François, DE CARLI Emilie, DEMOOR Charlotte, DUPLAN Mylène, FAUCHERE Magali, PELLIER Isabelle, PROUST-HOUDEMONT Stéphanie.
Toulouse : BERTOZZI-SALAMON Anne-Isabelle, BOULANGER Cécile, CASTEX M-Pierre, GAMBART Marion, PASQUET Marlène, PLAT Geneviève, SALEL Yu Jin
StDenis : BOHRER Sandrine, CHAMBON Fanny, HAMARCHI Hajar, JEHANNE Mathilde, REGUERRE Yves,
